# Detecting Mind Wandering: An Objective Method via Simultaneous Control of Respiration and Fingertip Pressure

**DOI:** 10.3389/fpsyg.2019.00216

**Published:** 2019-02-05

**Authors:** Yilei Zheng, Dangxiao Wang, Yuru Zhang, Weiliang Xu

**Affiliations:** ^1^State Key Laboratory of Virtual Reality Technology and Systems, Beihang University, Beijing, China; ^2^Beijing Advanced Innovation Center for Biomedical Engineering, Beihang University, Beijing, China; ^3^Department of Mechanical Engineering, The University of Auckland, Auckland, New Zealand

**Keywords:** sustained attention, mind wandering detection, simultaneous control, respiration, fingertip pressure, temporal synchronization

## Abstract

Mind wandering happens when one train of thought, related to a current undertaking, is interrupted by unrelated thoughts. The detection and evaluation of mind wandering can greatly help in understanding the attention control mechanism during certain focal tasks. Subjective assessments such as random thought-probe and spontaneous self-report are the ways previous research has assessed mind wandering. Here we propose a task in which participants are asked to simultaneously control respiration and fingertip pressure. They are instructed to click a force sensor at the exact moment of inhalation and exhalation of their respiration. The temporal synchronization between the respiratory signals and the fingertip force pulses offers an objective index to detect mind wandering. Twelve participants engaged in the proposed task in which self-reports of mind wandering are compared with the proposed objective index. The results show that the participants reported significantly more mind-wandering episodes during the trials with a larger temporal synchronization than they did during those trials with a smaller temporal synchronization. The findings suggest that the temporal synchronization might be used as an objective marker of mind wandering in attention training and exploration of the attention control mechanism.

## Introduction

Sustained attention, focusing on a target or a task and resisting the occurrence of unrelated thoughts, is a fundamental human ability that ensures effective cognitive processing (Raz and Buhle, 2006; Chun et al., 2011). However, our attentions are not always tied to ongoing events or to tasks we are performing. When we produce thoughts unrelated to an ongoing task, this is commonly referred to as mind wandering (Antrobus et al., 1970; Smallwood and Schooler, 2006). Several studies have associated mind wandering with a range of beneficial functions such as planning and creativity (Baird et al., 2011, 2012). Nevertheless, mind wandering’s correlation with costly outcomes such as driving accidents (Yanko and Spalek, 2013), working inefficiency (Farley et al., 2013; Seli et al., 2016), affective dysfunction (Smallwood et al., 2003; Killingsworth and Gilbert, 2010), and impaired performance in daily life (McVay et al., 2009) has received far more attention.

One example of involuntary mind wandering often occurs during meditation. During focused attention meditation, participants are required to maintain attention on specific content such as breathing or a candle flame. In the sustained breath awareness task, for example, participants need to focus continuously on their breath; if they realize the occurrence of mind wandering, they are instructed to reorient focus toward the breath (Braboszcz and Delorme, 2010; Hasenkamp et al., 2012). However, participants, especially novices, are generally unaware of mind wandering at the moment it occurs. It has previously been observed that meditation is a promising tool to improve attention span and reveal the attention control mechanism (Lutz et al., 2004, 2009; Tang et al., 2007; Brewer et al., 2011). Studies exploring the effects of intense meditation training on mind wandering have also suggested that meditation training reduces the susceptibility of minds to wander, subsequently leading to longer periods of meditative absorption and better attentional performance (Brandmeyer and Delorme, 2016; Zanesco et al., 2016). Accordingly, detecting mind wandering during meditation is a crucial and necessary step toward enhancing the effectiveness of attention training and may contribute to exploration of the neural mechanisms underlying the regulation of sustained attention (Schooler et al., 2011; Schmalzl et al., 2015).

Mind wandering has been mainly detected through two thought-report methods: discrete thought-probes (Allan Cheyne et al., 2009; Stawarczyk et al., 2011; Seli et al., 2013; Levinson et al., 2014) and spontaneous self-reports (Smallwood and Schooler, 2006; Braboszcz and Delorme, 2010). In the former, participants are randomly probed about their subjective attentional states; one of these ways is being asked to press buttons during a specific task. This method is easy to implement but valid only at the moment of the probe. It misses some vital information such as the time of alternating states, the starting moment, and the duration of a mind-wandering episode. In addition, the mental state of participants after a thought-probe cannot be assessed: whether they continue wandering, restart a new wandering, or refocus attention back to the task.

In spontaneous self-reports, participants are requested to note the moment they become aware of mind wandering. This method allows continuous tracking of mind wandering from the participant’s perspective. However, this tracking is subjective and limits the ability of researchers to maintain consistent evaluation among different participants. Furthermore, monitoring one’s own mind wandering is actually a task which may induce mind wandering (Bastian and Sackur, 2013; Vugt et al., 2015). Both methods have a fundamental flaw in that a participant’s mind wandering is evaluated solely by themselves, and that participants may not be aware when their attention drifts away.

To improve the aforementioned methods for tracking mind wandering, there have been extensive efforts made in pursuing objective measures or sensor-based metrics. Some of these measures are behavioral, including response time (RT) variability (Bastian and Sackur, 2013; Esterman et al., 2013; Rosenberg et al., 2013, 2015; Seli et al., 2013), increased error rate (McVay and Kane, 2012), and decreased comprehension (Smallwood et al., 2008; Schad et al., 2012). Other measures are electro-physiological and neurological, including increased galvanic skin response (Smallwood et al., 2007), pupil dilation (Smallwood et al., 2011; Wainstein et al., 2017), increased activity in the default mode and executive networks (Christoff et al., 2009), increased energy in theta and delta bands and decreased energy in the alpha and beta bands (Braboszcz and Delorme, 2010), and decreased amplitude of sensory-triggered ERP (Kam et al., 2011).

Nevertheless, since mind wandering is an inherently subjective experience, subjective feedback remains an important method for monitoring attentional state. In the studies using random thought-probes (Christoff et al., 2009; Seli et al., 2013), the measures within the few seconds preceding off-task reports and within the few seconds preceding on-task reports were contrasted. In the studies using spontaneous self-reports (Braboszcz and Delorme, 2010; Hasenkamp et al., 2012; Bastian and Sackur, 2013), the measures within the seconds preceding the report and within the seconds following the report were contrasted. Note that for the measures using self-reported mind-wandering episodes, participants must be alerted to the instruction that they should refocus attention to the task immediately after reporting mind-wandering episodes.

Different from previous studies on exogenous attention, this paper makes an attempt to explore the objective method for mind-wandering detection in an endogenous attention task. It should be clarified that mind wandering encompasses a broad range of phenomena and there is still not a uniform definition of mind wandering in previous studies (Seli et al., 2018). Mind wandering in our study is defined as task-unrelated thoughts.

This paper proposed a novel respiration–force coordinating task wherein participants were instructed to click a force sensor when they started to inhale and exhale. Respiratory signals, fingertip pressure, and self-reports of mind-wandering episodes were recorded during the task. The temporal synchronization between the respiratory signals and the fingertip force pulses was defined as an objective index to detect mind wandering. Experimental results based on 12 participants indicated that the participants reported significantly more mind-wandering episodes during the trials with a larger temporal synchronization than those trials with a smaller temporal synchronization. In addition, the durations of mind-wandering episodes in the task were estimated according to different task responses.

## Materials and Methods

### Participants

Twelve healthy students (four females; mean age = 24 ± 1.62 years, range = 22–27 years) from Beihang University participated in the study. All participants were right-handed and had no cognitive deficit disorders or somatosensory disorders. Notably, none of them had experience in meditation or related exercises. This study was approved by State Key Laboratory of Virtual Reality Technology and Systems of China. All the methods were performed in accordance with the relevant guidelines and regulations. All participants provided written informed consent prior to participation and each of them was paid ¥150 (about $23) upon completion of the experiment.

### Apparatus

**FIGURE 1:**
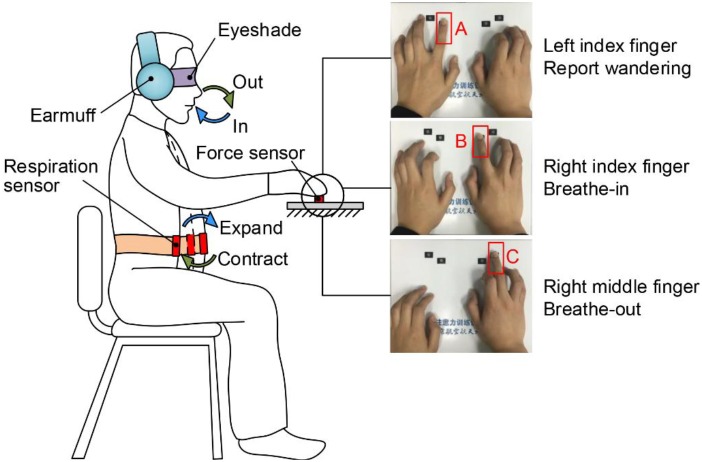
Experimental task. Participants sat in a chair in front of the fixed force-sensor plate, tied a respiration sensor belt to the abdomen, and wore an eyeshade and a pair of earmuffs. They clicked button B using the right index fingertip when beginning to breathe in and clicked button C using the right middle fingertip when beginning to breathe out. Button A was clicked by the left index fingertip to report mind wandering whenever the participants were aware of it. The data of fingertip force pulses and respiration were recorded in real-time.

A respiration sensor (used in combination with NeXus-10 Mark II, Mindmedia Inc., Netherlands) was tied to the participant’s abdomen to measure the respiratory signals. Three force sensors (A, B and C in Figure 1, FSG15N1A, Honeywell Inc., United States) were mounted on a fixed plate to measure the forces exerted by the left index, right index, and right middle fingertips, respectively.

### Experimental Task and Procedure

All participants conducted the respiration–force coordinating task in the experiment. The coordinating task involves synchronous controlling of fingertip force pulses and deep breathing. Deep breathing practices, which generally involve inhalation and exhalation of air at a slow rate that is different from regular breath cycles, form an integral component of many meditation programs (Brown and Gerbarg, 2009). A number of studies show that the deep breathing practice may result in marked beneficial effects across a variety of cognitive functions, including inhibitory control, working memory, and attention and emotion regulation (Busch et al., 2012; Brown et al., 2013; Vlemincx et al., 2016; Yadav and Mutha, 2016). Depending on the breathing pattern, deep breathing practices can take various forms (Brown et al., 2013); during the respiration-force coordinating task, the participants were instructed to keep a constant, slow, and deep diaphragmatic breathing rhythm with a brief pause following the inspiratory/expiratory period of each breathing cycle. Instead of requiring a specific respiration rate and depth, the participants were instructed only to breathe as slowly and deeply as possible to maintain comfort throughout the breathing cycle.

Figure 1 shows the procedure of the respiration–force coordinating task, during which participants sat in a chair in front of the force-sensor plate at a convenient height. They were required to click button B using the right index fingertip at the exact time when they started breathing in. Similarly, they clicked button C using the right middle fingertip when beginning to breathe out. The participants were asked to try their best to keep the temporal synchronization between the respiratory signals and fingertip pressure. Additionally, the participants were instructed to immediately report whenever they became aware of mind wandering. They did this by clicking button A with the left index fingertip and then refocusing attention back to the task.

Mind wandering during the task is defined as “failing to click any buttons when breathing in or out” or “clicking the wrong button, such as button C when breathing in or button B when breathing out.” These guidelines were instructed to all participants prior to the experiment. To prevent confusion, the participants were required to rest their fingers on the top of corresponding buttons before each session of the experiment so that they would not click a wrong button.

Before the experiment, participants were provided with written instructions and sufficient practice to make sure they were fully familiar with the experimental requirements. The whole experiment lasted 1 h and 15 min, split into four sessions of 15 min with 5-min breaks between two adjacent sessions. The participants were required to wear an eyeshade for eliminating external visual disturbance and a pair of head-mounted earmuffs for eliminating surrounding noise during the task.

### Accuracy

**FIGURE 2:**
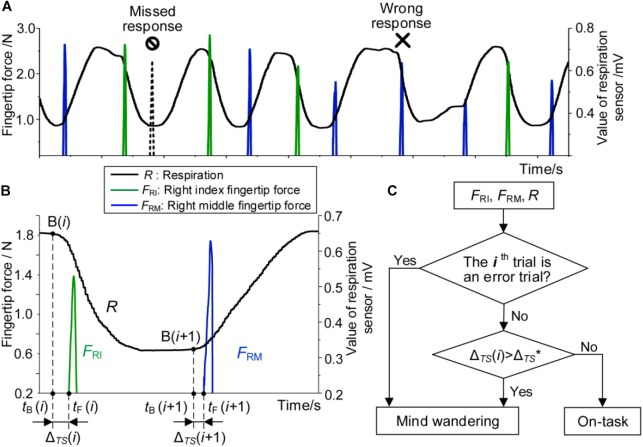
**(A)** Definition of error trials, including missed response and wrong response. **(B)** Δ_TS_ in two consecutive trials. **(C)** Model of detecting mind wandering. Error trials and correct trials with Δ_TS_ values larger than Δ_TS_^∗^ were considered “mind wandering.” Correct trials with Δ_TS_ values smaller than Δ_TS_^∗^ were considered “on-task.” Δ_TS_^∗^ was the median of Δ_TS_ for all correct trials in each session.

We defined each inhalation or exhalation within a respiratory cycle as a trial. Trials in which participants clicked the corresponding button correctly when they breathed in/out were considered correct trials. Two types of trials were considered error trials: (a) missed response, i.e., participants did not click any buttons during inhalation or exhalation phases within a respiratory cycle; (b) wrong response, i.e., participants clicked the wrong button (clicked button C when breathing in or clicked button B when breathing out), as shown in Figure 2A. Accuracy was defined as the percentage of correct trials over all trials in each session.

### Temporal Synchronization

In this work, the time difference between starting to breathe in/out and clicking the corresponding button was named the temporal synchronization (Δ_TS_). In the *i*th trial, the in-breath start moment was denoted by *t*_B_(*i*), as shown in Figure 2B. The moment when the force was greater than 0.2 N was defined as the button-click moment and denoted by *t*_F_(*i*) in Figure 2B considering the zero drift of the force sensors. Δ_TS_ of the *i*th trial was then computed as follows:

(1) $$\Delta_{\text{TS}}(i)=|t_{\text{B}}(i)-t_{\text{P}}(i)|$$

Given that the respiratory signals reached plateaus near to the peak and valley, calculating the end data points of the peak and valley plateaus [i.e., B(*i*) and B(*i*+1) in Figure 2B] is the key of the algorithm.

The algorithm steps were stated as follows. First, band-pass filter (0.5–100 Hz) and moving average filtering were used to remove some obvious noise. Second, we segmented the filtered data into segments of each respiratory cycle by setting the minimum interval among two adjacent peaks and minimum amplitude of peaks. So that the moments of the maximum points and minimum points in each data segment can be obtained. Last, for each data segment, least square method was used to obtain a straight line which passed the maximum or minimum point to fit the plateaus of peak or valley. The intersections of this straight line with the filtered signals were then calculated. Generally, more than one intersection would be obtained. However, the intersection with the maximum abscissa (i.e., the corresponding moment) was considered the end of the peak or valley plateau.

The accuracy of the algorithm for computing the temporal synchronization is about 50 ms, which meets our requirements for the subsequent analysis. In addition, it should be noted that Δ_TS_ of a missed response trial was treated as half of the duration of the associated respiratory cycle. Its value was much larger than that of correct trials. Δ_TS_ of a wrong response trial was, however, defined as the duration from the beginning of breathing-in/-out to the moment of clicking the wrong button. Its value was not necessarily larger than that of correct trials.

### Model of Detecting Mind Wandering

We hypothesized that the internal attentional states can be objectively reflected by external measurable signals including respiratory signals and fingertip pressure. All participants were provided sufficient practice prior to the formal experiment to ensure they were fully familiar with the respiration-force coordinating task. Considering that the task is simple enough for all participants and that they reported a good mental state during the whole experiment, it is reasonable to state that error trials and poor temporal synchronization (Δ_TS_(*i*) larger than Δ_TS_^∗^) reflect mind wandering.

We proposed an objective model to detect mind wandering during the task (Figure 2C). The inputs included the force of the right index fingertip *F*_RI_, the force of the right middle fingertip *F*_RM_, and the respiratory signal *R*. The output was the attentional state at each trial: “mind wandering” state or “on-task” state. Here we defined Δ_TS_^∗^ as the median of Δ_TS_ for all correct trials in each session. To compute Δ_TS_^∗^, the Δ_TS_ values of all correct trials were calculated and normalized by min–max normalization method (Shalabi et al., 2006) for each session of each participant. The Δ_TS_^∗^ of each session was then used to distinguish “mind wandering” state and “on-task” state. Those correct trials (Δ_TS_(*i*) > Δ_TS_^∗^) in each session were considered “mind wandering,” which represents a poor temporal synchronization between respiration and fingertip pressures signals. Other correct trials (Δ_TS_(*i*) ≤ Δ_TS_^∗^) with a good temporal synchronization were considered “on-task.”

### Estimating Duration of Mind Wandering

**FIGURE 3:**
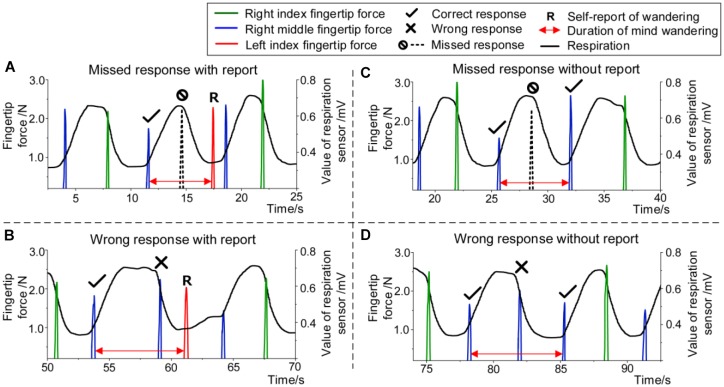
Estimating duration of mind wandering. For **(A)** missed response with self-report and **(B)** wrong response with self-report, the duration was considered from the end of the latest correct trial to the self-report. For **(C)** missed response without self-report and **(D)** wrong response without self-report, the duration was considered from the end of the latest correct trial to the beginning of the next correct trial.

The duration of mind-wandering episode for error trials was estimated as illustrated in Figure 3. For error trials with self-report, including missed response (Figure 3A) and wrong response (Figure 3B), the duration was determined from the end moment of the latest correct trial to the start moment of the self-report. As for missed response and wrong response without self-report (shown in Figure 3C,D), the duration was determined from the end moment of the latest correct trial to the start moment of the next correct trial.

## Results

### Overall Performance

**FIGURE 4:**
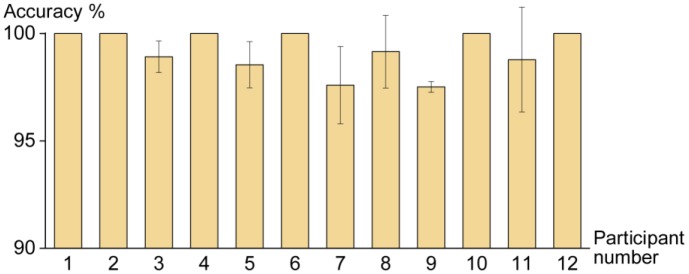
Mean accuracy of four sessions for all participants during the task. Error bars indicate ±*SD*.

The mean accuracy of four sessions for each participant is presented in Figure 4. All participants made more than 97.5% of correct trials on average. Half of the participants made no errors in all sessions.

**FIGURE 5:**
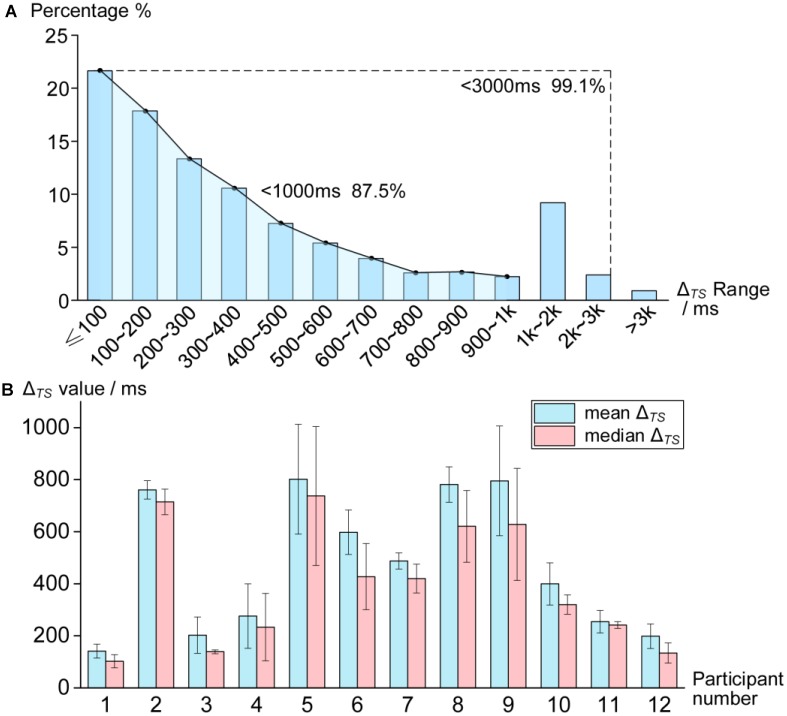
Overall performance of Δ_TS_. **(A)** Proportion of different Δ_TS_ ranges for all correct trials of four sessions. **(B)** Mean and median of Δ_TS_ for correct trials of four sessions for each participant. Error bars indicate ±*SD*.

To further assess the mean Δ_TS_ and the variability of Δ_TS_, we first calculated the proportion of different Δ_TS_ ranges for all correct trials (shown in Figure 5A). The results illustrated that the Δ_TS_ of 99.1% of correct trials was shorter than 3000 ms. Considering that such trials with Δ_TS_ exceeding 3000 ms may not be caused by participants, we excluded these trials when calculating the mean Δ_TS_ in the subsequent analysis. Moreover, the Δ_TS_ shorter than 1000 ms took up 87.5% of correct trials and the proportion of trials with smaller Δ_TS_ was even higher (Figure 5A). Together these findings demonstrated that participants performed accurate temporal synchronization in most trials.

The mean and median of Δ_TS_ for correct trials are displayed in Figure 5B. The results of paired *t*-test showed that the mean Δ_TS_ was significantly greater than the median Δ_TS_ (*t* = 5.247, *p* < 0.001). Therefore, the mean Δ_TS_ was not reasonable to evaluate participants’ performance because the overall performance would be underestimated. That was why we chose the median split analytical approach, defining Δ_TS_^∗^ as the median Δ_TS_, instead of the mean value of Δ_TS_ for all correct trials in each session.

### Detection of Mind Wandering

Table 1 lists the number of the detected error trials from all participants and the error trials without self-report (named as “missed-report”). The results showed that there were 26 error trials, and 8 of these error trials were missed-reports (caused by four participants).

**Table 1:** Number of trials in different conditions (Error or Correct trials, with or without self-report, Above or Below).

Participants number	Error trials				Correct trials			
	With self-report		Without self-report		With self-report		Without self-report	
	Above	Below	Above	Below	Above	Below	Above	Below
1	0	0	0	0	1	3	184	193
2	0	0	0	0	0	1	73	72
3	2	1	0	0	2	0	134	139
4	0	0	0	0	1	0	77	80
5	5	0	0	0	2	0	162	164
6	0	0	0	0	1	0	141	142
7	3	0	1	0	1	0	74	81
8	0	0	1	1	0	0	78	79
9	4	1	3	0	1	0	157	160
10	0	0	0	0	5	1	333	337
11	1	1	2	0	1	0	157	158
12	0	0	0	0	1	0	170	174
Total	15	3	7	1	16	5	1740	1779

*Above: Δ_TS_(i) > Δ_TS_^∗^; below: Δ_TS_(i) ≤ Δ_TS_^∗^.*

**FIGURE 6:**
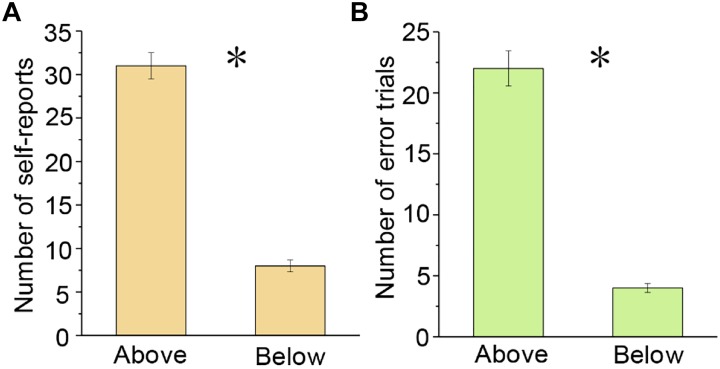
Number of self-reports and error trials during trials above and below the Δ_TS_^∗^. Participants **(A)** reported significantly more mind-wandering episodes and **(B)** made significantly more error trials during trials above the Δ_TS_^∗^ than those below the Δ_TS_^∗^. ^∗^*p* < 0.05. Error bars indicate ±*SD*.

In order to assess the effectiveness of Δ_TS_^∗^ in mind wandering detection, we counted and compared the number of self-reports during trials above the Δ_TS_^∗^ (Δ_TS_(*i*) > Δ_TS_^∗^) and that below the Δ_TS_^∗^ (Δ_TS_(*i*) ≤ Δ_TS_^∗^). Based on the data of the four columns of “Error trials-with self-report-Above,” “Error trials-with self-report-Below,” “Correct trials-with self-report-Above,” and “Correct trials-with self-report-Below” in Table 1, we analyzed the correlation between the number of self-reports and the Δ_TS_ values. One-way ANOVA on the number of self-reports showed that the participants reported significantly more mind-wandering episodes during trials above the Δ_TS_^∗^ than those below the Δ_TS_^∗^ [*F*(1,22) = 7.175, *p* < 0.05, Figure 6A].

In addition, we analyzed the correlation between the number of error trials and the Δ_TS_ values based on the data of the four columns belonging to “Error trials.” One-way ANOVA on the number of error trials showed that the participants made significantly more error trials during trials above the Δ_TS_^∗^ when compared with those below the Δ_TS_^∗^ [*F*(1,22) = 4.477, *p* < 0.05, Figure 6B]. These findings demonstrated that the participants were in a state of inattention during trials above the Δ_TS_^∗^, when they tended to report more mind-wandering episodes and make more error trials.

### Duration of Mind Wandering

**FIGURE 7:**
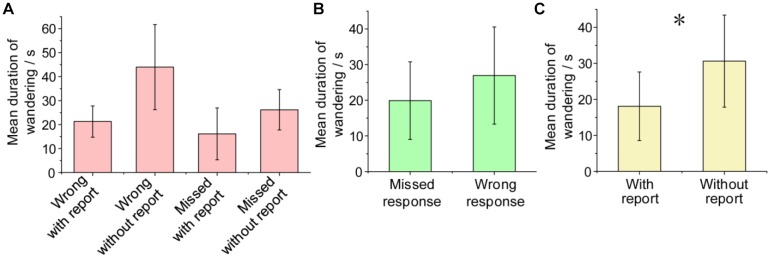
Mean duration of mind wandering for error trials in different conditions. **(A)** Mean duration of mind wandering from all participants, grouped by four conditions of trials. **(B)** Mean duration when participants made wrong responses was longer than that when they missed responses. **(C)** Mean duration for error trials without self-report was significantly longer than that with self-report. ^∗^*p* < 0.05. Error bars indicate ±*SD*.

We estimated the duration of mind-wandering episodes in the proposed task according to different task responses (see the section “Materials and Methods”). The duration of mind wandering for error trials in the task obtained from all participants varied across 3–56.5 s (mean 22.3 ± 11.3 s). Furthermore, Figure 7A shows the mean duration of mind wandering for error trials in different conditions (wrong response or missed response, with self-report or without self-report). Contrasts between the missed response trials and the wrong response trials showed that there was no significant difference between the mind-wandering duration when the participants made wrong responses (26.9 s, *SD* = 13.6) and that when they missed responses [19.9 s, *SD* = 10.9; one-way ANOVA, *F*(1,7) = 1.513, *p* > 0.05], as shown in Figure 7B. In addition, the mean mind-wandering duration for error trials without self-report (30.6 s, *SD* = 12.8) was significantly longer than that with self-report [18.1 s, *SD* = 9.5; one-way ANOVA, *F*(1,11) = 4.882, *p* < 0.05], as shown in Figure 7C.

## Discussion

Given that monitoring mind wandering during internally oriented states such as meditation is inherently subjective and thus notoriously difficult to measure, in this study we proposed a method for assessing mind wandering during an internally focused breath awareness task. This task requires participants to monitor the exact moment of inhalation and exhalation of their breath while simultaneously pressing a button at the moment of each individual inhalation and exhalation. As breathing is a naturally occurring phenomenon that continues without effort, the correspondence between the participants self-reports and the inspiratory/expiratory moment is a good indication of whether the participants are focused on the task or whether they have begun mind wandering. The experiment results support that the larger temporal synchronization between the respiratory signals and the fingertip pressures indicated the mind-wandering state. Together these results add to the growing list of objective measures used to detect mind wandering.

Several previous studies (Bastian and Sackur, 2013; Esterman et al., 2013; Rosenberg et al., 2013, 2015; Seli et al., 2013; Laflamme et al., 2018) highlight RT variability instead of raw RT (corresponding to Δ_TS_ in this paper) as an important indicator of attentional state in some focal tasks such as the Sustained Attention to Response Task (SART) (Helton et al., 2009). Using a go/no-go paradigm, SART requires participants to repetitively respond to the stimuli as quickly and as accurately as possible. During this task, deviant RTs, whether fast or slow, represent reduced attention to the task. Abnormally slow RTs may indicate reduced attention to the task or inefficient processing of the ongoing stream of visual stimuli thus requiring more time to accurately discriminate targets, while abnormally fast RTs may indicate premature or routinized responding and inattention to the potential need for response inhibition. Performance in SART is subjected to a speed–accuracy tradeoff resulting from strategy choices and from the failures of controlling motor reflexes (Dang et al., 2018). In our study, we used raw Δ_TS_ instead of Δ_TS_ variability as the index of attentional state. We considered the following aspects. First, the stimulus signal in our task is the onset of inhalation or exhalation in each respiratory cycle. Participants pay attention to their respiration and produce the fingertip force pulses, which are more related to endogenous attention rather than response inhibition. Therefore, we confidently hypothesize that participants can monitor their respiratory signals and produce correct fingertip force pulses with small Δ_TS_ easily. The speed–accuracy tradeoff does not need to be considered since the respiratory signals can be controlled by participants. Second, adopting Δ_TS_ variability (standard deviation or coefficient of variation of several trials) as the index of attentional state to our work is unreasonable since the duration of each trial is not constant.

Statistical results of Table 1 verify that the relatively large value of Δ_TS_ effectively reflects the occurrence of mind wandering in the respiration–force coordinating task. Whenever the participants made error trials or reported mind-wandering episodes, Δ_TS_ values exceeded the Δ_TS_^∗^ for most cases. Nevertheless, the Δ_TS_^∗^ is a relative value which varies between subjects and between sessions within-subjects. The Δ_TS_^∗^ depends on the Δ_TS_ values of all correct trials in each session. Thus, it may be hard to realize the real-time online detection of mind wandering via the method of Δ_TS_^∗^. One possible solution is determining a threshold for Δ_TS_ based on the absolute values of Δ_TS_. Of course, the Δ_TS_ threshold should be modified according to different participants and adjusted adaptively as the performance of task changes.

In addition, the proposed index Δ_TS_ cannot ensure uninterrupted monitoring of mind wandering (i.e., there is a blind window in the temporal domain). It can be evidenced by the contradiction between the small values of Δ_TS_ and self-reports as listed in Table 1. In these trials, participants produced a good temporal synchronization on the task but still reported a mind-wandering episode. The reason is that Δ_TS_ is only sampled and computed at the beginning of inhalation or exhalation. The detection of a brief mind-wandering episode in the duration between two adjacent sampling points may be missed through this method. In other words, the temporal resolution of the mind wandering detection is half of the respiratory cycle. One effective solution for improving the temporal resolution is to require participants to increase/decrease fingertip pressure along with the inhalation/exhalation process synchronously. By observing the synchronization between the temporal gradient of respiratory signals and that of fingertip pressure signals, a higher temporal resolution index could be devised to realize continuous monitoring of mind wandering.

It should be noted that the mind-wandering levels of error trials and of correct trials with poor temporal synchronization are considered different although they are both caused by mind wandering. This can be explained by the characteristics of the task. Given that the task is simple and all participants carried out adequate practice before the formal experiment, it is mind wandering that impairs a participant’s performance on the task. These impairments can be found in two results: error trials and increased Δ_TS_. In the error trials, the level of mind wandering was so deep that the participants completely forget to produce force pulses or produce a wrong force pulse. However, in the correct trials with large Δ_TS_ values, participants produced a correct response with a poor temporal synchronization. This level of mind wandering was relatively weak, or the duration of mind wandering was so short that the attention refocused on the task before the participants realized the occurrence of mind wandering. One possibility is that the participants distributed some attentional resources to other task-unrelated thoughts, which inevitably reduced the speed of force production, and thus led to the relatively large values of Δ_TS_.

Moreover, the duration of mind wandering in the respiration–force coordinating task fluctuated with in a range of 3–56.5 s according to different types of error trials (wrong response, missed response, with and without self-report). It must be noted that the estimation of mind-wandering duration in this paper is conservative. The estimation method is based on the assumption that mind wandering occurs immediately after the end of the latest correct trial and continues until the beginning of next correct trial or the self-report. The obtained duration of mind wandering also includes the interval from realizing mind wandering to clicking the button, and therefore is longer than the actual duration. Nevertheless, one advantage of the estimation method is that the duration can be computed separately according to different types of error trials, which may help to explore different types of mind-wandering episodes. The mind-wandering duration for the correct trials with poor temporal synchronization can also be estimated by this method; the duration is considered as the time span of consecutive trials with a Δ_TS_ value above the Δ_TS_^∗^.

A critical point worth noting is that the interpretation of results in this paper is based on a basic assumption – errors produced in the respiration–force coordinating task reflect mind wandering (more accurately, task-unrelated thoughts). This assumption is at least a fact for our relatively simple task, which can be validated by the data in Table 1 (18 mind-wandering episodes were reported following errors trials, while only 8 errors were produced in the absence of self-reports). It demonstrated that most participants are able to report their mind-wandering episodes when errors occurred during the task. However, those eight missed-reports may be attributed to two possibilities. One is that the mind wandering indeed happened and caused the error, but the participants did not notice the occurrence of mind wandering or they might have forgotten to report. The other possibility is that the participants produced wrong fingertip response pulses when they were not thinking about task-unrelated thoughts. Maybe there was a misalignment between perceptual and finger-controlling processes in the absence of task inattention and therefore the assumption we made in this paper may have potential limitation. Nevertheless, the interpretations of results in this paper are still valid since the absence of self-reports following error trials in the results was minor. Meanwhile, an experiment with a larger sample size is worthwhile and essential to further verify the effectiveness and robustness of Δ_TS_ on detecting mind wandering.

## Conclusion

The proposed respiration–force coordinating task provides an objective method to detect mind wandering in the endogenous attention task. The major difference from previous objective detection methods of mind wandering is that stimulus signals originate from internal human bodies (i.e., the onset of inhalation or exhalation in each respiratory cycle) rather than external visual or auditory presentations. The respiration–force coordinating task preserves the feature of internal focus guidance in meditation practices. It brings a promising solution for objective monitoring of mind wandering during meditation and attention training.

Based on the present work, one possible place for future research is applying the proposed method to online mind-wandering detection and reducing the frequency of mind-wandering episodes in attention training by alerting practitioners once their minds start wandering. Longitudinal studies are also expected to be performed in future research to explore whether long-term training of the respiration–force coordinating task could improve attention. Furthermore, the mechanism underlying the effect of the task on attention modulation could be revealed via neuroimaging methods.

## Data Availability Statement

The raw data supporting the conclusions of this manuscript will be made available by the authors, without undue reservation, to any qualified researcher.

## Author Contributions

YiZ and DW designed, performed, analyzed, and wrote up the research. YuZ and WX critically reviewed and edited the manuscript.

## Conflict of Interest Statement

The authors declare that the research was conducted in the absence of any commercial or financial relationships that could be construed as a potential conflict of interest.
